# Needs Must When

**Published:** 1920-09-04

**Authors:** 


### THE EDITOR'S LETTER-BOX.

**Correspondence on all subjects is invited, but we cannot in any way be responsible for the opinions expressed by our correspondents, who must give their name and address as a guarantee of good faith, but not necessarily for publication. Correspondents are reminded that brevity of style and conciseness of statement greatly facilitate early insertion.**

### "Needs Must When ——"

### "General Practitioner's" Reply to "Social Worker."

*To the Editor of* The Hospital.

Sir,—I am much obliged for your courtesy in forwarding me a copy of the contribution from a Social Worker [see p. 575], dealing with what I wrote recently in your columns. While recognising the earnestness and sincerity of "Social Worker," may I call his (or her) attention to one or two points? In the first place I did not write, nor did I mean, that those of the population for whom sexual abstinence is impossible consist wholly of men, though "Social Worker" assumes that I did; this disposes at once of the greater part of his (her) remarks. On the contrary, though I believe a higher percentage of men are in that category than of women, yet many women, from ladies of title to humbler ranks of society, have told me themselves with every semblance of conviction that they find continence impossible. If I were free to discuss professional confidences, I could give chapter and verse enough to convince anyone. The Church of England marriage service contains a well-known passage which seems to show that the divines who drew it up recognised the same state of things three centuries ago. It is because I hold to the opinion quoted by "Social Worker" in italics from my previous letter, and because the problems of venereal disease cannot be solved if that fact is blinked, that I ventured to write to you on the subject.

Next, I did not say that the "Christian conflict with the lower impulses is folly." I believe the conflict is hopeless in a percentage of cases, and I believe that in a further percentage victory in that conflict is bought at a heavy price in neuroses and psychoses; but I also believe that in many cases the victory is comparatively easy, and that even when it is difficult the menace of it to health is much less than that of venereal disease.

Next, I cannot admit that the analogy "Social Worker" draws between the tendency to incontinence and the tendency to theft and murder is either fair or complete. The gratification of the sexual impulse is not an anti-social deed, and therefore is nowhere regarded by the State as a crime; the other acts are subversive of the welfare of the herd, the State, or whatever name one chooses to signify the aggregations of individuals into which man bands himself.

The argument that "this assumption involves the prostitute" is "Social Worker's" deduction, not mine. I want facts as they are to be faced; deductions from them are more debatable. I have only to observe that "Social Worker's" own contribution contains evidence of widespread prostitution; and that nineteen centuries of Christianity in Europe have made as little impression on it as they have upon venereal diseases. The fact (as "Social Worker" believes it to be) that school girls practise prostitution surely goes to support my contention; for in the first place the working-class population was never so well able to feed its children properly as now, and in the second there is a Meals for School Children Statute which prevents any such child being exposed to starvation. Heaven forbid that I should minimise the evils of prostitution, or that I should hinder "rescue work," or sneer at the prostitute, or that I should discriminate between the gander and the goose: I hope I did none of these things. It is just because I, as a medical man, am only too well acquainted with the ravages of venereal disease that I want to see no stone left unturned to diminish it; and it is because one of the societies which is setting out to diminish it seems to me to be shutting its eyes to an essential factor in the problem that I wrote the letter I did.—Yours faithfully,

"General Practitioner."

